# No Accession-Specific Effect of Rhizosphere Soil Communities on the Growth and Competition of *Arabidopsis thaliana* Accessions

**DOI:** 10.1371/journal.pone.0027585

**Published:** 2011-11-15

**Authors:** Anna G. Aguilera, Adán Colón-Carmona, Rick Kesseli, Jeffrey S. Dukes

**Affiliations:** 1 Biology Department, University of Massachusetts, Boston, Massachusetts, United States of America; 2 Department of Forestry and Natural Resources and Department of Biological Sciences, Purdue University, West Lafayette, Indiana, United States of America; University of Tartu, Estonia

## Abstract

Soil communities associated with specific plant species affect individual plants' growth and competitive ability. Limited evidence suggests that unique soil communities can also differentially influence growth and competition at the ecotype level. Previous work with *Arabidopsis thaliana* has shown that accessions produce distinct and reproducible rhizosphere bacterial communities, with significant differences in both species composition and relative abundance. We tested the hypothesis that soil communities uniquely affect the growth and reproduction of the plant accessions with which they are associated. Specifically, we examined the growth of four accessions when exposed to their own soil communities and the communities generated by each of the other three accessions. To do this we planted focal accessions inside a ring of six plants that created a “background” soil community. We grew focal plants in this design in three separate soil treatments: non-sterile soil, sterilized soil, and “preconditioned” soil. We preconditioned soil by growing accessions in non-sterile soil for six weeks before the start of the experiment. The main experiment was harvested after seven weeks of growth and we recorded height, silique number, and dry weight of each focal plant. Plants grown in the preconditioned soil treatment showed less growth relative to the non-sterile and sterile soil treatments. In addition, plants in the sterile soil grew larger than those in non-sterile soil. However, we saw no interaction between soil treatment and background accession. We conclude that the soil communities have a negative net impact on *Arabidopsis thaliana* growth, and that the unique soil communities associated with each accession do not differentially affect growth and competition of study species.

## Introduction

Plant-soil feedbacks have received recognition as a driving mechanism behind plant abundance and rarity [1], [2]. Plant species and even genotypes [3], [4] supply soil communities with litter, decomposing roots and exudates that provide distinct combinations of substrates to unique microbial and macro-invertebrate communities [5], [6], [7]. These soil communities influence nutrient cycling, plant nutrient availability, disease protection, plant health, and plant growth, thereby creating plant-soil feedbacks [1], [8], [9], [10], [11], [12]. To date, research on plant-soil feedbacks has focused on successional dynamics and invasive species. A meta-analytical review by Kulmatiski et al. [11] provides support for the hypothesis that negative feedbacks among early successional species accelerate succession while positive feedbacks later in succession stabilize communities [11], [13], [14], [15]. A growing body of evidence suggests feedbacks influence some plant invasions [11], [16], [17], [18]. Reinhart and Callaway [19] showed the invasive plant *Centaurea maculosa* to have negative plant-soil feedbacks in its native soil, and positive feedbacks in soil from its invaded range in North America. The authors suggest that this escape from negative feedbacks and facilitation in the invaded range contributes to *Centaurea*'s invasive success. The review by Kulmatiski et al. [11] revealed that, while positive and negative plant-soil feedbacks have been reported in the literature, the majority of feedbacks were negative (70%), and annuals experienced greater feedback responses than did perennial species. Feedback experiments often assume that microbial soil communities that exert negative impacts do so directly via pathogens or by limiting nutrient acquisition. However, microbes can also significantly limit plant productivity by competing with plants for nutrients [20], [21].

While there are several mechanisms for soil mediated effects on plant growth, there is a large body of evidence of direct, microbially-mediated host-specific plant-soil feedbacks. Bartelt-Ryser et al. [22] showed that host-specific soil communities persisted in the soil even after host species removal. Further support comes from research identifying specific soil microbial communities in the rhizosphere, the area where the soil microbial community is influenced by plant roots. For example, *Cicer arietium*, *Brassica napus*, and *Sorghum bicolor* each host a distinct community of Eubacteria in their rhizosphere [23]. It has also been demonstrated that species respond uniquely to microbial inoculants. Westover and Bever [8] found that *Anthoxanthum odoratum* and *Panicum sphaerocarpom* responded differently to host-specific isolates of *Bacillus mycoides*. Each plant species preferred the bacteria cultivated by the other; *Anthoxanthum* had a more positive growth response to *Bacillus* cultivated on *Panicum*, and *Panicum* had a more positive response to *Bacillus* cultivated on *Anthoxanthum*. Molecular research has also determined that plant exudates can influence expression of genes in some plasmids [24]. Clearly, exploration of the very complicated set of feedbacks at play in the rhizosphere has only just begun.

While the list of species-specific plant-soil feedbacks has grown rapidly, little effort has gone into detecting whether unique feedbacks exist for different natural variants (ecotypes) within a species. While potentially more subtle, it seems likely that such ecotype-specific feedbacks could be widespread; ecotypes themselves are genetically distinct, geographically separated populations that are uniquely adapted to their place of origin. Recent research shows that different genotypes within a plant species cultivate unique bacterial communities. Schweitzer et al. [4] have shown that intraspecific differences in microbial community composition between genotypes of *Populus* sp. are greater than interspecific differences. Using the model system *Arabidopsis thaliana* (hereafter referred to as *Arabidopsis*), Micallef et al. [3] discovered that different accessions (ecotypes) of *Arabidopsis* generate unique and reproducible bacterial communities in their rhizosphere. In addition, Bressan et al. [25] showed that a transgenic *Arabidopsis* plant line overproducing glucosinolates, secondary metabolites known for their antimicrobial properties, displayed distinct root exudate profiles and rhizobacterial communities (bacterial communities associated with plant roots). This implies that even slight genetic differences can significantly alter the chemistry of the rhizosphere and have a meaningful effect on the microbial communities associated with a given accession. Further, recent work by Biedrzycki et al. [26] describes how *Arabidopsis* is able to recognize self and kin (of same accession) plants via direct interaction with root exudates. They show that accessions exposed to a “stranger's” root exudates grew longer lateral roots than those that were exposed to “sibling” exudates. Different accessions of *Arabidopsis* have also been shown to have significantly different competitive effects and responses to competition with other accessions and species [27], [28]. However, the extent to which this is due to plant morphology as opposed to soil feedbacks is unknown.

We hypothesized that accession specific soil communities would each have unique effects on plant growth and competition. We tested this hypothesis with four *Arabidopsis* accessions. We grew each alone (solo), as a focal plant grown with background competitors from its own accession (genetic monoculture), and as a focal with each of the other three accessions as background competitors. Competition experiments were replicated in soil that was sterile, non-sterile, or that had been “preconditioned” with *Arabidopsis* plants. We expected that focal plant growth would vary depending on the ecotype of background competitors. We also expected that the rhizosphere communities of the different competitors would differentially affect focal plant growth. We predicted that these plant-soil feedbacks would be negative. Further, we anticipated that intra- accession feedbacks would be the strongest, and that plants exposed to “other” accessions would exhibit a “release” from co-evolved microbial species. Due to the a limited amount of time for competing plants to establish and generate rhizosphere communities that are in contact with focal plant root systems, we anticipated that differences in soil communities' effects would be small. We expected the soil communities in the preconditioned soil to be established at the start of the experiment. Therefore, we expected the effect of competing accessions to be greatest in the preconditioned soil treatment. To determine the degree to which nutrient depletion affected plants in the preconditioned soil we performed a “follow-up” experiment that included a fertilization treatment of the preconditioned soil.

## Methods

### Study species

*Arabidopsis thaliana* is an annual weed in the Brassicaceae that is commonly used in genetic and molecular studies. *Arabidopsis* is an ideal species with which to study the impact of soil feedbacks on competition for two reasons. First, Micallef et al. [3] have shown that different accessions of *Arabidopsis* produce distinct and reproducible rhizosphere bacterial communities, with significant differences in both species composition and relative abundance. Second, *Arabidopsis'* small size and short life cycle allow for extensive replication and relatively short experimental durations.

### Competition experiment

We used four *Arabidopsis* accessions: Columbia (Col; USA), Cape Verde Islands (Cvi, Cape Verde), Landsberg *erecta* (*Ler*; Germany), and Rld (Rld-1; Russia). We selected these accessions to develop maximally different rhizobacterial communities as shown by Micallef et al. (2009). We sterilized all seeds in a 33% bleach solution, and thoroughly rinsed with sterile water. We then stratified all seeds in sterile water at 4°C for 48 hours before planting.

**Figure 1:**
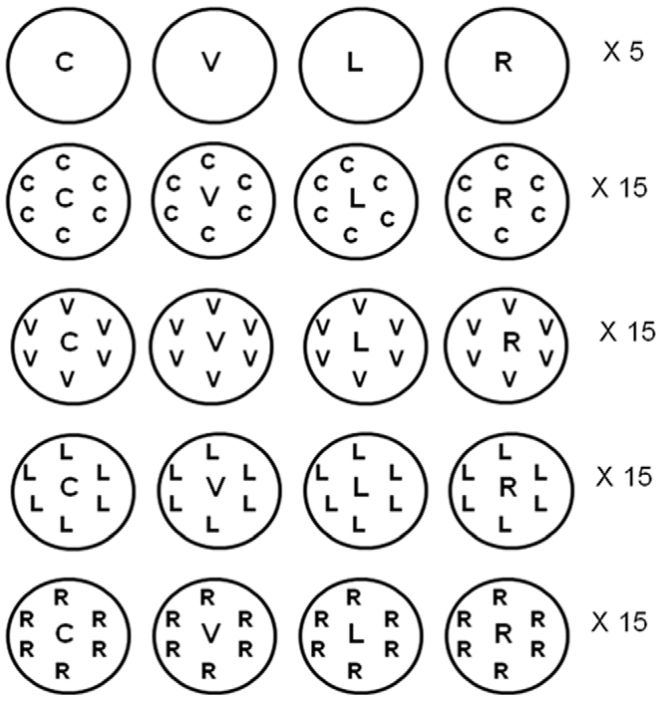
Schematic diagram of the experimental design. Each circle represents an experimental treatment. Letters represent individual plants. C = Col; V = Cvi; L = *Ler*; R = Rld.

We grew a single “focal” plant of each accession in competition with the same ecotype and with each of the other three accessions (Fig. 1). We planted 12 “background” seeds in a ring around the edge of a 5 cm “cone-tainer” (Stuewe & Sons, Inc., Tangent, Oregon). We thinned background plants to 6 plants per pot a week after germination. One week after planting the background seeds, we planted 3 focal seeds in the center of each pot. After 5–7 days we thinned to 1 focal plant per pot. In addition we planted “solo” treatments with focal plants of each accession grown alone in a cone-tainer to determine the effect of competition (Fig. 1). We replicated the competition treatments 15 times, and the solo treatments 5 times, using a block design, with solo treatments in blocks 1–5. We replicated this design in three soil treatments: sterile, non-sterile, and preconditioned.

We collected soil from fallow fields at the University of Massachusetts Suburban Experiment Station in Waltham, Massachusetts, USA (Lat = 42°23.1′N; Long = 71°12.9′W). We sieved the soil on site (to remove stones and macro-invertebrates), homogenized the soil, and stored it at 4°C until planting. All cone-tainers received a mixture of 10 g of field soil and 35 g of sterilized commercial potting soil. This mixture was established by Micallef et al. [3] as optimal for *Arabidopsis* growth. We sterilized the potting soil by autoclaving it in batches 1–2 cm deep. We autoclaved each batch twice, for 1 hr each time, with a 72 hour resting period in between (modified from Trevors 1996). We created a sterile soil treatment by sterilizing field soil with the same technique and adding it to the sterilized potting soil. To create the non-sterile treatment, we mixed non-sterile field soil with intact microbial communities from the field with sterilized potting soil. For this treatment we considered the non-sterilized field soil as a microbial inoculum from which the *Arabidopsis* plants could cultivate their unique soil communities. We created a “preconditioned” soil treatment by growing each of the four ecotypes in the non-sterile mix (non-sterile field soil with sterilized potting soil) for 6 weeks prior to the start of the experiment. We preconditioned the soil in large shallow greenhouse flats using a plant to soil ratio equal to that of pots in the final competition experiment. We kept the soils conditioned with different accessions separate from one another. We removed preconditioning plants from the soil and collected and mixed soils to homogenize soil within ecotype and removed all visible roots. We stored the soils for 24 hours in closed buckets before planting. When planting, we matched the preconditioned soil with the background accession.

We grew all plants with a 12-hour light regime in a plant-growth room, with 95 µmol PAR/m^−2^. After 7 weeks (6 weeks of focal plant growth) we harvested all the plants. We measured main inflorescence height, leaf number, and silique number for each focal plant. We then dried the aboveground tissue of all focal and background plants at 65°C and weighed it.

### Preconditioning follow-up

After harvesting the competition experiment we collected the soil from the preconditioned treatment for a preconditioning “follow-up” experiment. Keeping the soil preconditioned with the 4 different accessions separate, we split the soil into 3 soil treatments: fertilized, sterilized, and untreated. We gave the fertilized treatment 15 ml of half strength Scotts® Miracle Grow® (N, PO_4_, K, B, Cu, Fe, Mn, Mo, Zn, EDTA) solution, sterilized the sterile treatment as described for the competition experiment, and left the soil unchanged for the untreated treatment. In each pot we planted 3 seeds of each accession, with 6 replicates of each treatment. After 5 weeks we harvested all plants, dried the tissue at 65°C and weighed it.

### Measurements of Competition Severity and Statistical Analyses

We calculated the absolute severity of competition (ASC; [29], [30]) for each treatment as:
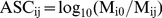
(1)Where M_i0_ is the mass of accession i in the solo treatments, and M_ij_ is the mass of accession i (focal) when grown with background competitors of accession j. ASC provides a measure of the effect of competition from the background plants on the performance of the focal plant. We determined ASC using blocks 1–5, which contained solo treatments.

To analyze the competition experiment data we used a series of ANCOVAs with soil (sterile, non-sterile, preconditioned), focal accession (Col, Cvi, *Ler*, Rld), and background accession (Col, Cvi, *Ler*, Rld) as fixed effects, and background plant mass as a covariate, with a separate ANCOVA for each response variable. Additionally we performed a MANCOVA with all response variables and report Pillai's Trace, Wilks' Lambda, Hotelling's Trace, and Roy's Largest Root test statistics. To compare individual means we used post-hoc Tukey's HSD tests. Finally, to detect treatment effects on ASC we used a 3-way ANOVA with soil, focal accession, and background accession as fixed factors. We log-transformed all mass, silique number, and height data for the competition, follow-up, and precondition experiments to meet assumptions of parametric tests. To analyze the preconditioned follow-up experiment we first calculated the response variable as average mass per plant of a given accession in each pot (statistical analyses obtained identical results when the response variable was total mass per pot). We then used an ANOVA with soil treatment (fertilized, sterilized, untreated), plant accession (Col, Cvi, *Ler*, Rld), and preconditioned soil type (Col, Cvi, *Ler*, Rld) as fixed effects. Again, to compare individual means we used a post-hoc Tukey's HSD test.

## Results

### Competition experiment

**Figure 2:**
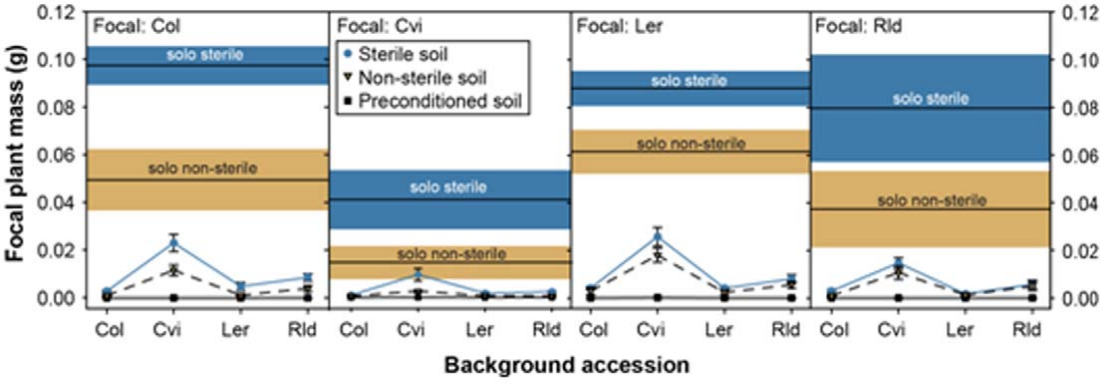
Mean mass (±SE) of focal plants Col, Cvi, *Ler*, Rld with each of the four background accessions, in all three soil types (• sterile soil, ▾ non-sterile soil, ▪ preconditioned soil). Mean mass of sterile, and non-sterile, solo plants are given by lines surrounded by dark gray and light gray regions depicting ±SE. Means and SE for solo plants are shown for reference and are the same for all background accessions because they grew without background plants (N = 15).

**Figure 3:**
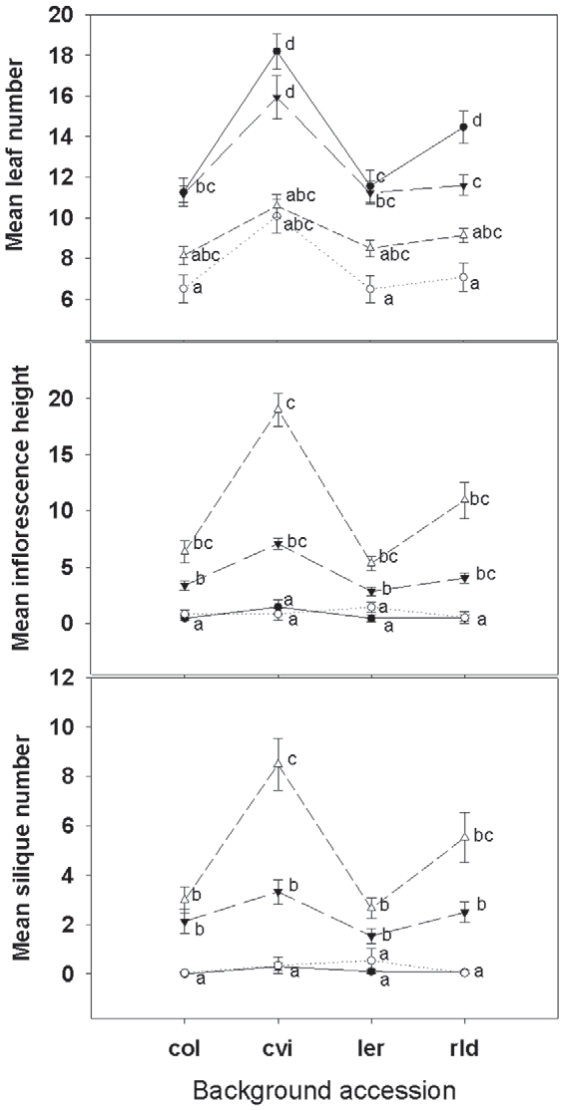
Mean leaf number, inflorescence height, and silique number (±SE) showing significant interactions between focal accession (• Col, ○ Cvi, ▾ *Ler*, Δ Rld) and background accession. Letters indicate significant differences between treatments using a Tukey-Kramer post-hoc paired comparison adjustment (N = 15).

**Figure 4:**
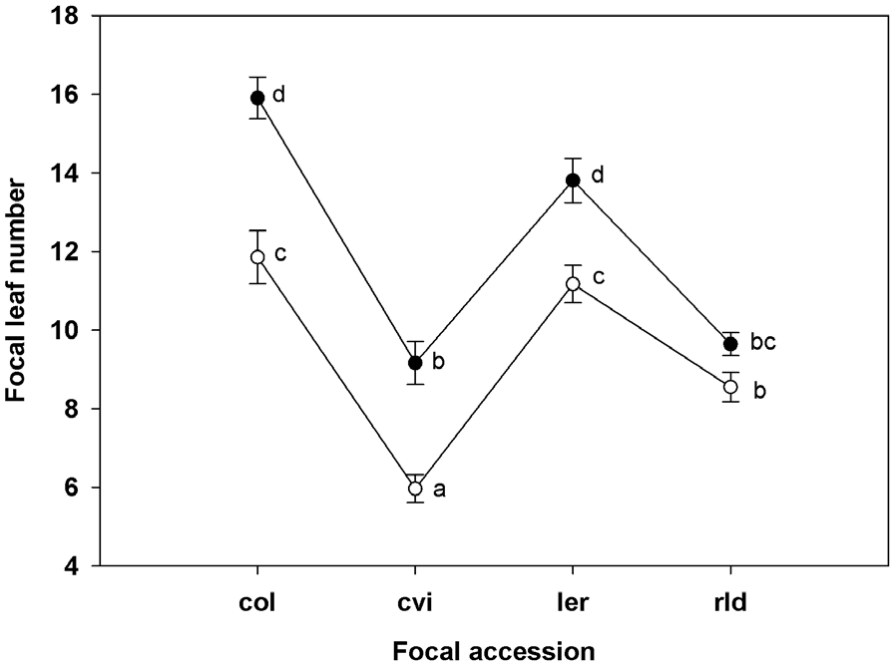
Mean number of leaves (±SE) showing significant interaction between focal accession and soil treatment for focal plant (○ non-sterile soil, • sterile soil). Letters indicate significant differences between treatments using a Tukey-Kramer post-hoc paired comparison adjustment (N = 15).

Focal plants in sterile soil grew larger than those in non-sterile soil, and plants in both of these treatments grew significantly larger than those in the preconditioned treatment (Table 1). All plants grown in preconditioned soil were extremely small and their inclusion in ANOVAs led to ecologically meaningless, and statistically unreliable, significant interactions (due to violations in assumptions for parametric tests). Therefore, we included only sterile and non-sterile soil treatments in further analyses. For all response variables (mass, leaf number, inflorescence height, and silique number), the main effects of soil, focal accession, and background accession were all significant (Table 2). Plants grown in sterile soil were always larger, had more leaves, greater height, and more siliques. Col and *Ler* accessions had the greatest mass, and Cvi the least. Consequently, focal plants with Cvi background competitors grew larger than those with the other three accessions as background plants (Fig. 2.). All focal accessions had more leaves when grown with Cvi background plants. Rld and *Ler* also showed increased inflorescence height and silique number with Cvi background plants. However, this is not surprising given that Col and Cvi bolted later and had shorter inflorescences and fewer siliques in all treatments. Relative to Col and *Ler*, Rld was also a weak competitor. Rld background plants had a similar effect to Cvi background plants on focal plant growth (Fig. 3, Table 2.). Col had the greatest total increase in leaves with sterilization, while Cvi had the greatest relative increase in leaf number (Fig. 4, Table 2). Plants grown alone (solo) in sterile soil were larger then solo plants in non-sterile soil (one-way ANOVA *F_2,59_* = 158.049, *P*<0.001). Consistent with the univariate test results, all the MANCOVA test statistics were significant for soil, plant accession, background accession, soil×focal accession, and focal accession×background accession interaction (all test statistics P<0.01). Therefore not only did individual response variables significantly differ, but we also saw significant separation of the multivariate group centroids with our treatments.

**Table 1:** Mean mass (±SE) of focal plants in sterile, non-sterile, and preconditioned soil (N = 15).

	Preconditioned	Non-sterile	Sterile
Mean Mass (g^−4^)	0.90	44.00	78.00
(SE)	(0.0000058)	(0.00067)	(0.00048)

**Table 2:** Three way ANOVA for all response variable (mass, leaf number, inflorescence height, and silique number), using the sterile and non-sterile soil treatments.

	Mass	Leaves	Height	Silique number
Soil	**<0.001** 62.811_(1,424)_	**<0.001** 69.023_(1,422)_	**0.007** 7.232_(1,421)_	**0.044** 4.077_(1.421)_
Focal accession	**<0.001** 48.502_(3,424)_	**<0.001** 88.123_(3,422)_	**<0.001** 215.496_(3,421)_	**<0.001** 207.403_(3,421)_
Background accession	**<0.001** 72.886_(3,424)_	**<0.001** 2.176_(3,422)_	**0.002** 4.883_(3,421)_	**<0.001** 8.198_(3,421)_
Soil × Focal	0.560 0.688_(3,424)_	**0.014** 3.586_(3,422)_	0.826 0.299_(3,421)_	*0.057* 2.527_(3,421)_
Soil × Background	0.988 0.044_(3,424)_	0.791 0.348_(3,422)_	0.561 0.686_(3,421)_	0.293 1.246_(3,421)_
Focal × Background	0.973 0.307_(9,424)_	**0.017** 2.282_(9,422)_	**0.005** 2.674_(9,421)_	**0.043** 1.955_(9,421)_
Soil × Focal × Back	0.214 1.339_(9,424)_	0.161 1.459_(9,422)_	0.959 0.347_(9,421)_	0.872 0.503_(9,421)_

P values (P<0.05 in bold, 0.05<P<0.1 in italics). Below are F values (numerator df, denominator df).

**Figure 5:**
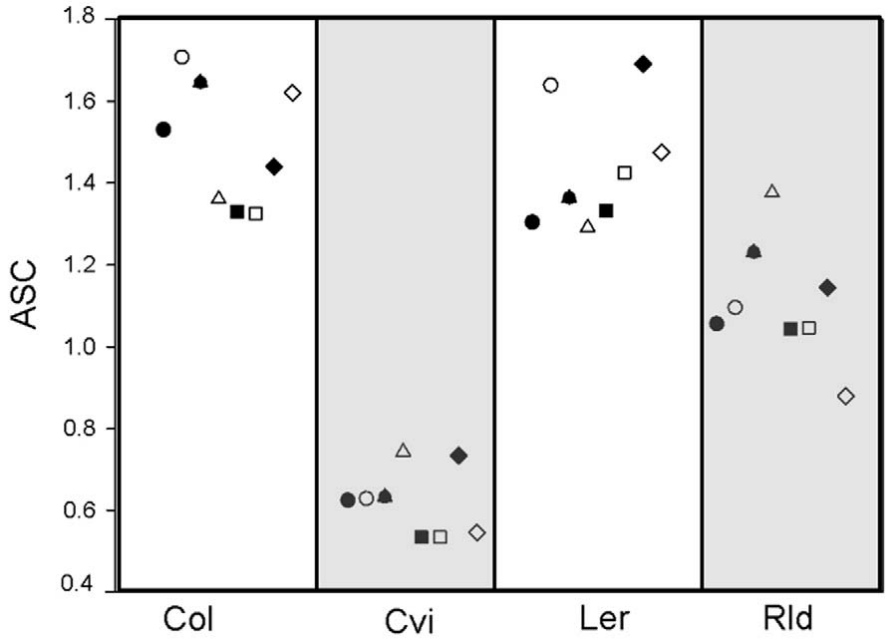
Mean ASC values experienced by focal plants (• sterile Col, ○ non-sterile Col, ▴ sterile Cvi, Δ non-sterile Cvi, ▪ sterile *Ler*, □ non-sterile *Ler*, ♦ sterile Rld, ◊ non-sterile Rld) with each background accession (N = 5).

Neither soil treatment nor focal accession affected ASC. However, background accession did affect ASC (3-way ANOVA for ASC background accession, *F_3,132_* = 17.149, *P*<0.001). ASC values for Cvi were smaller than the other three accessions, and Rld had smaller ASC values than Col and *Ler* accessions (Fig. 5).

### Preconditioning follow-up

**Figure 6:**
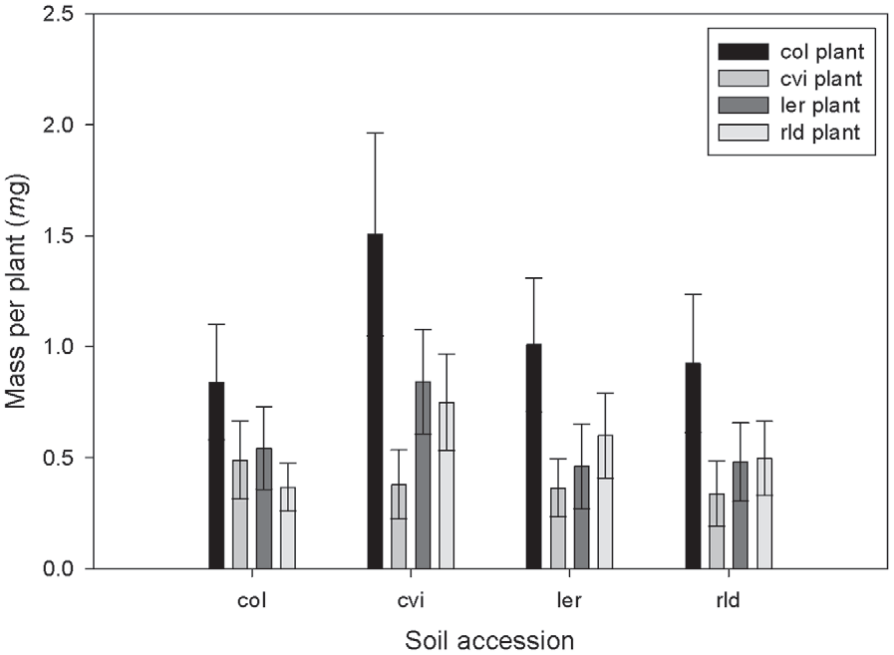
Mean mass (±SE) of focal plants in the preconditioned follow-up in each of the four preconditioned soils (Col, Cvi, *Ler*, Rld)(N = 15).

Due to plant mortality we used the average mass of each accession per pot rather than total mass in each pot. To justify this we performed a linear regression that showed no correlations between the number of plants per accession per pot and the average mass of plants (r^2^ = 0.003, *P* = 0.351). Plants grew largest in soil treated with fertilizer, but also showed increased size in sterilized soil relative to the untreated soil (3-way ANOVA for mass: soil treatment *F_2,220_* = 84.248, *P*<0.001; Tukey's HSD, fertilized-sterilized, *P*<0.001; fertilized-untreated, *P*<0.001; sterilized - untreated, *P*<0.001). Soil accession also had a significant effect on plant size (3-way ANOVA for mass: soil accession, *F_3,220_* = 4.224, *P* = 0.006). Plants grown in Cvi preconditioned soil grew larger than those grown in Col or Rld preconditioned soil (Fig. 6; Tukey's HSD: Cvi-Col, *P* = 0.028; Cvi-Rld, *P* = 0.008). Finally, Col plants were larger than the Cvi and *Ler* accessions, and Rld plants were larger than Cvi plants (3-way ANOVA for mass: plant accession, *F_3,220_* = 7.669, *P* = 0.000; Tukey's HSD: Col-Cvi *P* = 0.000; Col-*Ler P* = 0.032; Cvi-Rld *P* = 0.050).

## Discussion

We saw no evidence to support our hypothesis that the soil communities of different *Arabidopsis* accessions differentially affect *Arabidopsis* growth. However, as we expected, focal plant growth did vary with different accessions of background competitors. The preconditioned treatment did provide exaggerated results, though not in the manner that we predicted. Rather than intensifying any soil mediated effects on focal plant growth, the preconditioned soil suppressed the growth of all of the plants in the cone-tainer. Although there was no accession-specific soil effect on plant growth, the soil community did negatively influence plant growth, with all accessions experiencing the same positive effect of sterilization.

Consistent with the literature on competition between *Arabidopsis* accessions [28], [22] we saw morphologic and phenotypic differences among *Arabidopsis* accessions, and a significant effect of accession on competition and our plant response variables. Col and *Ler* focal plants were larger and had more leaves than the more delicate Cvi and Rld accessions (Figs. 3, 4). Focal plants with Col and *Ler* in the background had smaller mass, shorter inflorescences, fewer leaves and fewer siliques than plants with Cvi or Rld accession in the background (Figs. 3,4). This is unsurprising as the Col line was selected for vigor from a Landsberg (Lan) population in a greenhouse setting. Rld began bolting and flowering before the other three accessions, and *Ler* followed as the next to bolt and flower. As a result at harvest these two focal accessions had the tallest inflorescences and greatest number of siliques (Fig. 3). However, these morphological differences were probably affected by the timing of harvest. Had the plants each grown to senescence we might have seen a different pattern. We chose the harvest time because older leaves decompose rapidly as plants senesce, affecting the leaf count and mass of the plant.

We saw no evidence that *Arabidopsis* accessions exerted stronger intra- rather than inter-accession competition (Fig. 5). Rather, all accessions grew best when competing with Cvi, the smallest of the four accessions in the experiment. Although they used different accessions of *Arabidopsis*, Cahill et al. [27] also saw competitive strengths vary among accessions. Using multiple accessions of *Arabidopsis*, Bossdorf et al. [28] tested for interspecific competition between *Arabidopsis* and *Senecio vulgaris* and *Anagallis arvensis*. Similar to our results on intraspecific competition, Bossdorf et al. [28] saw differences among *Arabidopsis* accessions in their competitive effects on neighboring plants as well as in their responses to neighboring plants. In addition to the increasing evidence that *Arabidopsis* competitive ability varies with accession, Weltzin et al. [31] found that invasion of *Arabidopsis thaliana* communities by the congener *Arabidopsis suecica* was unaffected by the genetic diversity (number of different accessions) in the community. Weltzin et al. [31] planted seeds of *Arabidopsis suecica* into already established *Arabidopsis thaliana* communities composed of 1, 2, 4 and 8 accessions. The accession composition of each *Arabidopsis thaliana* community was generated by drawing accessions at random (without replacement) from a pool of 23. They found that density of the individuals in the *Arabidopsis thaliana* community had a strong effect on the size and reproductive potential of the invader. It is clear the accession of *Arabidopsis* is an important factor in determining individual's competitive success, and that the density of *Arabidopsis* populations is a driving factor behind their population dynamics.

Soil treatment also had an effect on focal plant growth. Plants grown in sterile soil were larger than those grown in non-sterile soil, while the plants grown in preconditioned soil were drastically smaller (Table 1). To minimize differences in nutrient availability between our sterile and non-sterile treatments we used sterile potting soil as the great majority of the substrate in both the sterile and non-sterile soil treatments. The only difference in the treatments was the treatment of the field soil that was mixed in. Therefore we assume that differences in soil treatments are primarily due to differences in the soil community.

The negative feedback we observed in our sterile treatment is in agreement with the review by Kulmatiski et al. [11] that showed most feedbacks to be negative in direction. Kulmatiski et al. [11] compared the range of plant-soil feedback effect sizes from their meta-analysis to those of meta-analyses of pathogenic fungi [32], leaf-litter addition [33], seed limitation, seed feeders, above ground herbivores, total herbivores, viruses, leaf chewers, root feeders [34], and soil warming [35]. In each case the effect sizes for plant-soil feedbacks were similar to or larger than those in the other meta-analyses. However, they found plant-soil feedback effect sizes to be smaller than those of plant competitors, plant diversity [32], below ground herbivores, pathogens, and nematodes [34]. While grasses have exhibited the most negative feedbacks [11] *Arabidopsis* as an herbaceous annual would also be expected to have more negative plant-soil feedbacks than trees and other perennial species. These findings are in keeping with the hypothesis that negative feedbacks increase the rate of successional replacement, with early colonizers experiencing the most negative feedback.

In the follow-up experiment, plants grew largest in the soil treated with fertilizer, but also grew larger in sterilized soil. Further, the soil accession (accession with which the soil was preconditioned for the original experiment) had an effect on plant size. Nutrient depletion probably contributed to the stunted growth of plants in the preconditioned soil. However, the preconditioned follow-up experiment also provided evidence of soil communities influencing plant growth, in both general and accession-specific ways.

The preconditioning soil treatment revealed that there were soil-mediated influences on plant growth in this experiment. The preconditioning follow-up experiment suggested that nutrient depletion and microbial interactions could have been responsible. Below-ground interactions have been highlighted by recent related work that showed genetic differences in *Arabidopsis* accessions influence growth and intra-specific competition via root exudates [26]. Biedrzycki et al. [26] saw increased lateral root development when *Arabidopsis* accessions were exposed to “stranger” (other accessions') root exudates in comparison to exposure to “sibling” (same accession) exudates. While this type of below-ground interaction may have affected our competition results, the extreme results of the preconditioned treatment suggest that it is also likely that competition for nutrients between plants and microbes affected our results. In the past it was thought that plants only used inorganic N that was left by microbial N mineralization. However, it is now acknowledged that plants, including *Arabidopsis* [36], are also able to use organic N, that they compete directly with microbes for this resource [37] and that the ability of plants to compete with microbes is critical for plant N acquisition and subsequent growth [38]. Bardgett et al. [21] showed that, in a temperate grassland, microorganisms were able to sequester the majority of both organic and inorganic N sources, thereby limiting plant productivity. Furthermore, stimulation of the microbial community by glucose addition increases microbial acquisition of N and causes a decrease in plant productivity [39]. We assume that our preconditioned soil had a higher microbial density, and therefore the severe nutrient limitation faced in the plants in this treatment was made more severe by plant-microbe competition for N. Increased growth of plants after both fertilization and sterilization of this soil is further evidence of the effect of this competition. There are several reasons why we may have seen an effect of preconditioning accession on plant size in our follow-up experiment. It may be further evidence of microbial interactions influencing plant growth, indicating that the Cvi generated microbial community had the weakest effect on plant growth. However, it may also be an indirect effect of nutrient depletion by preconditioning plants; because the Cvi are the smallest plants it is possible that the original Cvi plants that preconditioned the soil used the least amount of nutrients, leaving more available for subsequent plantings to utilize. Additionally it is possible that the Cvi preconditioning plants encourage less microbial growth, which in turn would mean less competition for nutrients for the subsequent plantings.

This experiment found clear differences in competitive ability among different accessions of *Arabidopsis*, and found that *Arabidopsis* accessions were consistently negatively affected by their soil communities. We did not isolate the mechanism for these negative effects; nor can we distinguish between the effects of pathogenic microbes and plant-microbe competition for nutrients. We found no evidence that accession-specific rhizobacterial communities differentially influence plant growth and competition. It is possible that the unique rhizobacterial communities are a non-adaptive consequence of accession specific exudates. However, we performed this experiment using a single soil inoculate that was foreign to each accession. Therefore it remains possible that the soil communities of each accession may respond differently in “home” vs. “foreign” soil environments in a manner that has consequences for plant growth.
